# Residential Proximity to Major Roadways and Prevalent Hypertension Among Older Women and Men: Results From the Chinese Longitudinal Healthy Longevity Survey

**DOI:** 10.3389/fcvm.2020.587222

**Published:** 2020-11-17

**Authors:** Yao Yao, Kaixi Cao, Kehan Zhang, Tinglong Zhu, Dahai Yue, Hao Zhang, Jim Zhang, Xurui Jin, Yi Zeng

**Affiliations:** ^1^Center for Healthy Aging and Development Studies, National School of Development, Peking University, Beijing, China; ^2^Center for the Study of Aging and Human Development and Geriatrics Division, Medical School of Duke University, Durham, NC, United States; ^3^Global Health Research Center, Duke Kunshan University, Suzhou, China; ^4^Department of Health Policy and Management, Fielding School of Public Health, University of California, Los Angeles, Los Angeles, CA, United States; ^5^Department of Healthcare Policy and Research, Weill Cornell Medicine, New York, NY, United States; ^6^Global Health Institute and the Nicholas School of Environment, Duke University, Durham, NC, United States

**Keywords:** proximity to major roads, traffic related air pollution, blood pressure, hypertension, older adults, CLHLS

## Abstract

**Background and Objectives:** Prior studies suggested that residential proximity to major roadways was associated with increased risks of cardiovascular diseases in developed countries, for which one explanation is that road proximity could heighten the risks of hypertension. However, the association of residential distance to major roadways with hypertension is still unclear in low- and middle-income countries (LMICs) with levels of air pollution and socioeconomic development distinctively different from developed countries.

**Methods:** We derived data from the eighth wave of the Chinese Longitudinal Healthy Longevity Survey, a nationwide prospective cohort. The present study included 12,881 individuals older than 65 years (mean age, 85.2 ± 11.7 years) with 55.8% of them being female. We ascertained the residential proximity to major roadways based on self-reports and hypertension was defined as systolic blood pressure ≥140 mm Hg or diastolic blood pressure ≥90 mm Hg. We then used logistic regression to examine the association between residential distance to major roadways and hypertension.

**Results:** The odds ratios (ORs) of hypertension for participants living 50 to 100, 101 to 200, and ≥200 meters from major roads were 1.17 [95% confidence interval (95% CI) = 1.02–1.33], 1.21 (95% CI = 1.05–1.41), and 1.22 (95% CI = 1.10–1.34), respectively, compared to those living within 50 m (*P*_for trend_ < 0.001). Significant effects of modifications from socioeconomic status and accessibility to health care resources were observed (*P*s for interaction < 0.05). Compared to living within 50 m from a major roadway, the ORs of hypertension for living ≥50 m were higher in manual/agricultural workers, low-education groups, participants without household ventilation, and participants lacking in health education and health care resources. We observed considerable variations across geographic regions with the association in question attenuating in Eastern China but remaining significant in other regions.

**Conclusion:** Residential proximity to major roadways was associated with lower odds of hypertension among older adults in China. The utility of residential proximity to major roadways as a marker of increased risks of hypertension and cardiovascular diseases may need to be revisited in LMICs.

## Introduction

An increasing body of evidence indicates that in developed countries, such as the United States and Canada, residential proximity to major roadways was associated with higher prevalence of coronary heart disease (1), higher risk of acute myocardial infarction (2), and higher risk of cardiovascular-related mortality (3) and stroke (4). One potential explanation for such association is that living near major roadways increases the risk of hypertension, which in turn increases the risks of cardiovascular diseases. However, the association between residential distance to the nearest major roadway and hypertension was still understudied, and most prior studies were conducted among populations living in developed countries (5, 6). Considering the different residential choices and living environments between developed countries and low- and middle-income countries (LMICs), as well as the high disease burden of hypertension in LMICs (7, 8), there is an urgent need for studying the relationship between residential distance to major roadways and hypertension in LMICs to provide an evidence-based intervention for a more efficient and effective allocation of medical resources.

Living close to major roadways brings intense long-term exposure to traffic-related air pollutants and noise, each of which may be associated with higher blood pressure (9, 10). In addition to the effects from the living environments it brings, residential proximity to major roadways is also a multiple exposure that includes different socioeconomic status (SES), living arrangements, and lifestyles (11, 12). Prior studies suggested that its association with hypertension varies substantially by regions in the United States, with hazard ratios for women living ≤ 50 m from a major roadway being 1.61 [95% confidence interval (95% CI) = 1.18–2.20] in the West, 1.51 (95% CI = 1.22–1.87) in the Northeast, 0.89 (95% CI = 0.70–1.14) in the South, and 0.94 (95% CI = 0.75–1.19) in the Midwest, compared to those who live beyond 1,000 m from a major roadway (6). It is possible that different socioeconomic factors that come with geographical variations may play an important role in shaping the association between proximity to major roadways and hypertension. Varying so substantially even within developed countries by different regions, the association of residential proximity to major roadways with hypertension and its possibility of being a potential risk factor need to be studied and validated more rigorously in regions outside the developed world.

The present study has vital public health implications in studying the health impacts of physical living environments on hypertension in LMICs because of their fast speed of urbanization and high burden of cardiovascular disease. Accordingly, we derived data from the Chinese Longitudinal Healthy Longevity Survey (CLHLS), a nationwide longitudinal cohort, to examine the association between residential distance to major roadways and hypertension among Chinese older adults 65 years or older, adjusted for a number of potential confounders.

## Materials and Methods

### Study Population

The present study uses data from the 2018 wave of CLHLS, which is a longitudinal study since 1998 with follow-up surveys every 2 to 3 years. The CLHLS surveys were conducted in randomly selected counties and cities in China, which accounted for half of the counties and cities in 23 of 31 provinces covering >85% of China's population. Based on gender and place of residence (i.e., living in the same street, village, city, or county) for a given centenarian, randomly selected octogenarians and non-agenarians were also sampled. This matched recruitment procedure resulted in an oversampling of the oldest old and older men. In the CLHLS, a weight of age-sex urban/rural residence in the sample with the distribution of the total population in the sampled 22 provinces was employed to reflect the unique sampling design. More details of this survey have been published elsewhere (13).

**Figure 1:**
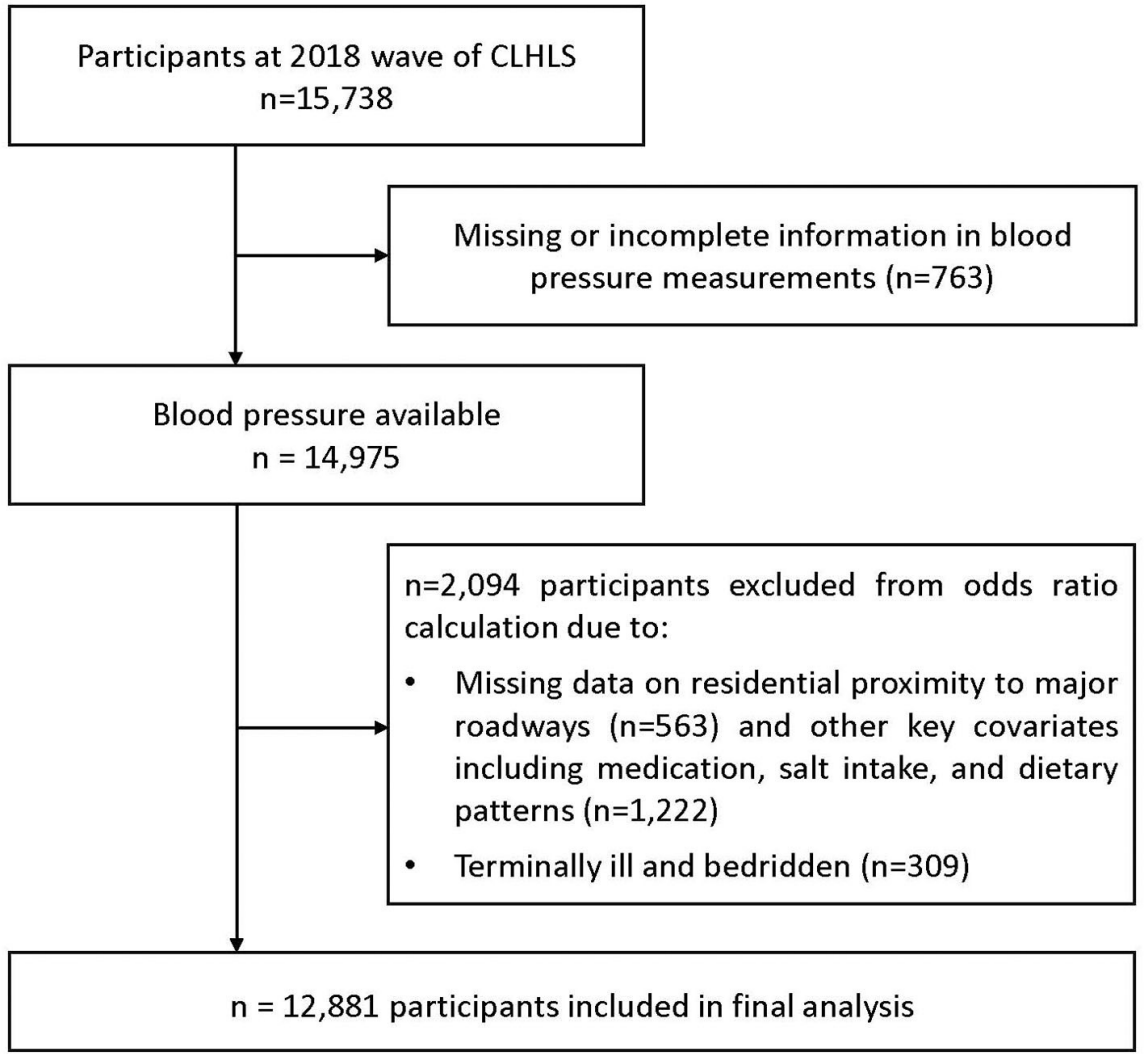
Derivation of the study population from participants of the Chinese Longitudinal Healthy Longevity Survey.

In the 2018 wave of CLHLS, the self-reported residential proximity to major roadways was first collected. After excluding 2,857 participants because of missing data on blood pressure and self-reported residential proximity to major roadways, the analytical sample included 12,881 participants older than 65 years (Figure 1). The demographic character of the analysis sample and total sample did not show significant difference (Supplementary Table 2).

### Residential Proximity to Major Roadways Information

Self-reported information on residential proximity to major roadways was collected through the face-to-face interview by trained research staff with more than 12 years' education. A 5-option question was used to categorize their current residential proximity to the nearest major roadways (unit in meter): <50, 50 to 100, 101 to 200, 201 to 300, and >300 (14). The self-reported distance was further categorized into <50, 50 to 100, 101 to 200, and >200 with sufficient sample size in each group, and prior evidence suggested that those living farther than 200 m from the major roadway may not be impacted by the traffic-related air pollutants from the heavy traffic (15).

### Measurement and Calculation of Blood Pressure

After participants had rested for at least 5 min, research assistants took two measurements of blood pressure on the right arm by mercury sphygmomanometer (upper arm type; Yuyue, Jiangsu, China) at the heart level. For bedridden participants, blood pressure measurements were obtained in a recumbent position. The mercury sphygmomanometer must be calibrated before each measurement. Systolic blood pressure (SBP) and diastolic blood pressure (DBP) were calculated as the average of the two measurements taken for an individual. We defined hypertensive participants if they met any of the following criteria as previous study: (1) SBP ≥140 mm Hg, a DBP ≥90 mm Hg; (2) current treatment with antihypertensive drugs in participants with a self-reported history of diagnosed hypertension (5–7). Severe hypertension was defined as SBP ≥180 mm Hg or DBP ≥120 mm Hg (16). Pulse pressure (SBP–DBP) and mean arterial pressure [((2 × DBP) + SBP)/3] were also calculated.

### Covariates

Covariates were chosen as potential confounders between exposures and outcomes or predictors of outcomes. We included the following covariates in our study: age, sex, marital status, occupation before retirement, education level, residence, smoking status, alcohol consumption, physical activity, salt intake, dietary diversity, family income, participants' expectance of the health care education, indoor cooking ventilation, cook fuel choice, body mass index (BMI), city population, participation of medical insurance, depressive symptom, cognitive impairment, activity of daily living (ADL), chronic conditions, and geographical region.

Age was calculated according to self-reported birth date, based on Chinese lunar calendar dates, and converted to Georgian calendar dates. We classified marital status into two categories: currently married and living with spouse as married and widowed/separated/divorced/never married/married but not living with spouse as not married. We divided residence into urban and rural areas based on governmental administrative categories. We used the schooling year to evaluate education level (<6 vs. ≥6 years). Smoking status was dichotomized as “non-current or non-smoker” vs. “current smoker;” a similar approach was taken to define the alcohol consumption and physical activity. Dietary diversity was calculated by intake frequency of eight food (vegetables, fruits, legumes and their products, nuts, meat, eggs, fish, and dairy and its products) and dichotomized as high vs. low with the cutoff of 6 (17). Family annual income was classified as <10,000 yuan, 10,000 to 30,000 yuan, and ≥30,000 yuan, and we dichotomized the family annual income into <10,000 yuan and ≥10,000 yuan in the subgroup analysis. Participants' expectance of the health care education was self-reported and dichotomized as “yes” and “no.” Household ventilation was classified as three categories: none, mechanical, and natural ventilation (by window or door opening). Primary cooking fuel choice was dichotomized into clean fuel (electricity, gas, solar energy) vs. polluted fuel (charcoal, firewood/straw). BMI was calculated as weight in kilograms divided by height in meters squared. City population was classified into four groups according to the permanent population: >8 million, 8 to 5 million, 5 to 3 million, and <5 million. Participation of two kinds of insurance included urban employee/resident medical insurance and new rural cooperative medical insurance. Depressive symptoms were measured by the 10-item Center for Epidemiologic Studies Depression Scale, and a cutoff of 10 was used to define a binary variable of depression episode based on previous studies (18). Cognitive function was assessed using Mini-Mental State Examination (MMSE), and a global score <24 indicates cognitive impairment (19). ADL was assessed by Katz Index, and we defined disability as needing personal assistance in performing one or more of the five essential activities (bathing, transferring, dressing, eating, and toileting) or being incontinent (20, 21). Common chronic diseases including diabetes, cancer, stroke, and cardiovascular disease were collected as self-reports. We considered geographical region on the basis of residential address to account for differences in regional economic developments, as well as social cultures: Northern China (Beijing, Tianjin, Hebei, Shanxi, Shandong, Liaoning, Jilin, and Heilongjiang provinces), Eastern China (Shanghai, Anhui, Shanxi, Zhejiang, Fujian, and Jiangsu provinces), Southern China (Guangdong, Guangxi, Henan, Hainan, Hubei, and Hunan provinces), and Western China (Sichuan, Chongqing, and Shaanxi provinces). All data were collected by trained interviewers through home interviews.

### Statistical Analysis

Baseline characteristics were presented as mean (continuous variables) or frequency distribution (categorical variable) by categories and binarized (<50 vs. ≥50 m) of residential proximity to major roadways. Analysis of variance for continuous variables and χ^2^ tests for categorical variables were applied to compare the differences among the categories. The association of residential proximity to major roadways with hypertension was assessed using multivariable logistic regression, and its association with SBP, DBP, pulse pressure, and mean arterial pressure was assessed using linear regression. We used four models: Model 1: adjusted for age, gender, and sampling weight; Model 2: additionally adjusted for residence (rural/urban), education level, marital status, occupation, lifestyle factors (smoking status, drinking status, dietary diversity, appetite, meat consumption, and physical activity), and BMI on the basement of Model 1; Model 3 additionally adjusted for indoor cooking ventilation, cooking fuel, family annual income, city population, participants' expectance of the community to provide health care education, geographical region, and participation of two kinds of medical insurance (urban employee/resident medical insurance, new rural cooperative medical insurance) on the basement of Model 2; and Model 4 further adjusted for depressive symptom, cognitive impairment, ADL, and four kinds of self-reported diseases (diabetes, heart disease, stroke, and cancer).

### Effect Modification

We dichotomized residential proximity to major roadways at a cutoff point of 200 m. We examined whether the association of residential proximity to major roadways with hypertension differed by several predefined factors: sex, age (<80 vs. ≥80 years old), occupation before retirement, residency, city population, education years, physical activity, cooking fuel choice, expectance of the community to provide health care education, family income, indoor cooking ventilation, depressive symptoms, and ADL. The significance of multiplicative interactions between dichotomized residential proximity to major roadways and other variables was tested by cross-product terms in the models.

### Sensitivity Analysis

We conducted several sensitivity analyses to check the robustness of the main results. First, we considered severe hypertension (SBP of 180 mm Hg or higher or a diastolic pressure of 120 mm Hg or higher) as outcome. Second, we excluded the participants who took antihypertensive drugs. Third, we excluded those who have changed address in 5 years. Fourth, we excluded those who have severe cognitive impairment (MMSE <19), which might derive substantial recall bias (22). Fifth, we replicated the main analysis by residency with substantial difference of road planning between rural and urban area. A two-tailed *P* < 0.05 was considered statistically significant. All analyses were performed using STATA version 14.0 (Stata Corp, College Station, TX, United States).

## Results

### Baseline Characteristics

The presented study included 12,881 participants older than 65 years. Demographic characteristics by category are shown in Table 1, and the distributions of hypertension, blood pressure, mean arterial pressure, pulse pressure, awareness of hypertension, and antihypertensive drug therapy are presented in Table 2. The mean age of participants was 85.2 (SD = 11.7) years, and the prevalence of hypertension was 63.1%. Participants living farther away from the major roadways are more likely to be older, with higher blood pressure, mean arterial pressure, and pulse pressure; living in rural areas; used to be manual and agricultural workers; with lower SES including education and income; using more polluted household fuel; not adopting indoor ventilation; having no medical insurance; and with worse lifestyle including lower dietary diversity, lower physical activity levels, and higher alcohol consumption (Tables 1, 2). The trend remained in binary analyses with dichotomized proximity of road to <50 and ≥50 m (Supplementary Table 1).

**Table 1:** Characteristics of 12,881 participants aged over 65 years old and older in the Chinese longitudinal healthy longevity survey.

**Characters**	**Distance to the major roadway**					***P*-value**
	** <50**	**50–100**	**101–200**	**>200**	**Total**	
N	2,352	1,899	1,262	7,368	12,881	
Age, years, mean (SD)	84.6 (11.6)	85.2 (11.7)	85.6 (11.7)	85.4 (11.8)	85.2 (11.7)	0.013
Sex, *n* (%)						0.73
Male	1,050 (44.6)	837 (44.1)	574 (45.5)	3,236 (43.9)	5,697 (44.2)	
Female	1,302 (55.4)	1,062 (55.9)	688 (54.5)	4,132 (56.1)	7,184 (55.8)	
Education, *n* (%)						<0.001
None (0 year)	1,342 (57.1)	991 (52.2)	637 (50.5)	4,432 (60.2)	7,402 (57.5)	
Primary school (1–6 years)	443 (18.8)	349 (18.4)	266 (21.1)	1,441 (19.6)	2,499 (19.4)	
Middle school or higher (>6 years)	567 (24.1)	559 (29.4)	359 (28.4)	1,495 (20.3)	2,980 (23.1)	
Occupation before retirement, *n* (%)						<0.001
Manual and agricultural workers	1,706 (72.5)	1,132 (59.4)	737 (58.4)	5,675 (77.0)	9,250 (71.8)	
White-collar workers	646 (27.5)	767 (40.6)	525 (41.6)	1,693 (23.0)	3,631 (28.2)	
Residence, *n* (%)						<0.001
Urban	1,375 (58.5)	1,266 (66.7)	828 (65.6)	3,552 (48.2)	7,021 (54.5)	
Rural	977 (41.5)	633 (33.3)	434 (34.4)	3,816 (51.8)	5,860 (45.5)	
Marital status, *n* (%)						0.27
Currently married and living with spouse	1,381 (58.6)	1,164 (61.3)	753 (59.7)	4,471 (60.7)	7,769 (60.3)	
Others^a^	971 (41.3)	735 (38.7)	509 (40.3)	2,897 (39.3)	5,112 (39.7)	
Dietary diversity^b^, *n* (%)						<0.001
High	1,809 (76.9)	1,356 (71.4)	896 (71.0)	5,793 (78.6)	9,854 (76.5)	
Low	543 (23.1)	543 (28.6)	366 (29.0)	1,575 (21.4)	3,027 (23.5)	
Tobacco smoking, *n* (%)						0.46
Current	378 (16.1)	278 (14.6)	182 (14.4)	1,105 (14.7)	1,943 (15.1)	
Not current	1,974 (83.9)	1,621 (85.4)	1,080 (86.6)	6,263 (85.3)	10,938 (84.9)	
Alcohol consumption, *n* (%)						0.22
Current	323 (13.7)	249 (13.1)	178 (14.1)	1,090 (14.8)	1,840 (14.3)	
Not current	2,029 (86.3)	1,650 (86.9)	1,084 (85.9)	6,278 (85.2)	11,041 (85.7)	
Physical activity, *n* (%)						<0.001
Yes	807 (34.3)	672 (35.4)	449 (35.6)	2,059 (27.9)	3,987 (31.0)	
No	1,545 (65.7)	1,227 (64.6)	813 (64.4)	5,309 (72.1)	8,894 (69.0)	
Appetite, *n* (%)						0.20
Salty	1,865 (79.2)	1,549 (81.7)	1,003 (79.8)	5,948 (81.1)	10,365 (80.3)	
Others^c^	487 (20.8)	350 (18.3)	259 (20.2)	1,420 (18.9)	2,516 (19.7)	
Meat consumption, *n* (%)						0.46
Almost every day	1,793 (77.1)	1,444 (76.7)	990 (78.8)	5,636 (77.4)	9,863 (77.1)	
Rarely or never	527 (22.9)	422 (23.3)	259 (21.2)	1,662 (22.6)	2,870 (22.9)	
Fuel choice, *n* (%)						<0.001
Clean	1,734 (73.7)	1,515 (79.8)	983 (77.9)	4,776 (64.8)	9,008 (69.9)	
Polluted	618 (26.3)	384 (20.2)	279 (22.1)	2,592 (35.2)	3,873 (30.1)	
Family annual income, yuan, *n* (%)						<0.001
<10,000	591 (25.1)	382 (20.1)	271 (21.5)	1,966 (26.7)	3,210 (24.9)	
10,000–30,000	392 (16.7)	301 (15.9)	169 (13.4)	1,432 (19.4)	2,294 (17.8)	
>30,000	1,369 (58.2)	1,216 (64.0)	822 (65.1)	3,970 (53.9)	7,377 (57.3)	
Indoor cooking ventilation, *n* (%)						<0.001
None	202 (8.6)	126 (6.6)	91 (7.2)	706 (9.6)	1,125 (8.7)	
Mechanical	1,037 (44.1)	1,026 (54.0)	727 (57.6)	2,722 (36.9)	5,512 (42.8)	
Window	1,113 (47.3)	747 (39.3)	444 (35.2)	3,940 (53.5)	6,244 (48.5)	
BMI (kg/m^2^), mean (SD)	22.3 (3.9)	22.4 (3.9)	22.5 (3.9)	22.1 (3.8)	22.3 (3.9)	<0.001
City population, *n* (%)						<0.001
>8 million	419 (17.8)	519 (27.3)	406 (32.2)	1,299 (17.6)	2,643 (20.5)	
5–8 million	1,107 (47.1)	874 (46.0)	496 (39.3)	3,614 (49.0)	6,091 (47.3)	
3–5 million	503 (21.4)	263 (13.8)	211 (16.7)	1,423 (19.3)	2,400 (18.6)	
<3 million	317 (13.5)	239 (12.6)	145 (11.5)	1,014 (13.8)	1,715 (13.3)	
Geographical region^d^						<0.001
Northern China	493 (21.0)	487 (26.6)	353 (28.0)	1,735 (23.5)	3,068 (23.8)	
Eastern China	581 (24.7)	523 (28.5)	360 (28.5)	2,046 (27.8)	3,510 (27.2)	
Southern China	919 (39.1)	613 (32.3)	392 (31.1)	2,622 (35.6)	4,546 (35.3)	
Western China	353 (15.0)	272 (14.3)	153 (12.1)	947 (12.9)	1,725 (13.4)	
Urban employee/resident medical insurance, *n* (%)						<0.001
Have	553 (23.5)	580 (30.5)	396 (31.4)	1,364 (18.5)	2,893 (22.5)	
Do not have	1,799 (76.5)	1,319 (69.5)	866 (68.6)	6,004 (81.5)	9,988 (77.5)	
New rural cooperative medical insurance, *n* (%)						<0.001
Have	1,470 (62.5)	962 (50.7)	621 (49.2)	5,128 (69.6)	8,181 (63.5)	
Do not have	882 (37.5)	937 (49.3)	641 (50.8)	2,240 (30.4)	4,700 (36.5)	
Expectance of the community to provide health care education, *n* (%)						0.43
Yes	1,397 (59.4)	1,139 (59.8)	842 (66.7)	4,675 (63.4)	8,053 (62.5)	
No	955 (40.6)	760 (40.2)	420 (33.3)	2,691 (36.6)	4,826 (37.5)	
Cognitive impairment^e^, *n* (%)						0.61
No cognitive impairment	1,439 (61.2)	1,139 (60.0)	776 (61.5)	4,422 (60.0)	7,776 (60.4)	
With cognitive impairment	913 (38.8)	760 (40.0)	486 (38.5)	2,946 (40.0)	5,105 (39.6)	
Activity of daily living, *n* (%)						<0.001
No activity of daily living	1,647 (70.0)	1,308 (68.9)	862 (68.3)	5,350 (72.6)	9,167 (71.2)	
With activity of daily living	705 (30.0)	591 (31.1)	400 (31.7)	2,018 (27.4)	3,714 (28.8)	
Depressive symptom^f^, *n* (%)						0.0017
No depressive symptom	1,089 (46.3)	864 (45.5)	555 (44.0)	3,164 (42.9)	5,672 (44.0)	
With depressive symptom	1,263 (53.7)	1,035 (54.5)	707 (56.0)	4,204 (57.1)	7,209 (56.0)	

a *“Others” include widowed, separated, divorced, and never married*.

b *Dietary diversity was calculated by intake frequency of eight food (vegetables, fruits, legumes and their products, nuts, meat, eggs, fish, and dairy and its products) and dichotomized as high vs. low with the cutoff of 6*.

c *“Others” include bland, sweet, spicy, and raw food*.

d *Northern China includes Jilin, Liaoning, Heilongjiang, Beijing, Tianjin, Shanxi, Hebei, and Shandong province. Eastern China includes Shanghai, Anhui, Shanxi, Zhejiang, Fujian, and Jiangsu province. Southern China includes Guangdong, Guangxi, Henan, Hainan, Hubei, and Hunan province. Western China includes Sichuan, Chongqing, and Shaanxi province*.

e *Cognitive impairment was defined as MMSE score lower than 24*.

f *Depressive symptom was defined by Center for Epidemiologic Studies Depression Scale score no fewer than 10*.

**Table 2:** Distribution of hypertension, blood pressure, mean arterial pressure, pulse pressure, awareness of hypertension, antihypertensive drug therapy according to the distance to the nearest major roadway.

**Characters**	**Distance to the major roadway**					***P***
	** <50**	**50–100**	**101–200**	**>200**	**Total**	
Hypertension^a^						0.009
Without	1,014 (43.1)	746 (39.3)	481 (38.2)	2,872 (39.0)	5,113 (39.7)	
With	1,338 (56.9)	1,153 (60.7)	781 (61.9)	4,496 (61.0)	7,768 (60.3)	
Severe hypertension^b^						<0.001
Without	1,987 (84.5)	1,628 (85.7)	1,085 (86.0)	5,971 (81.0)	10,671 (82.8)	
With	365 (15.5)	271 (14.3)	177 (14.0)	1,397 (19.0)	2,210 (17.2)	
Aware of hypertension^c^						<0.001
Not aware	485 (36.2)	389 (33.7)	234 (30.0)	1,772 (39.9)	2,880 (37.1)	
Aware	853 (63.8)	764 (66.3)	547 (70.0)	2,724 (60.1)	4,888 (62.9)	
Take the antihypertensive medicine						
No	746 (49.4)	696 (55.4)	509 (59.0)	2,396 (52.1)	4,347 (52.8)	<0.001
Yes	765 (50.6)	560 (44.6)	353 (41.0)	2,205 (47.9)	3,883 (47.2)	<0.001
Systolic blood pressure, mmHg	138.1 (20.3)	137.6 (20.00)	137.6 (19.6)	140.3 (21.1)	139.2 (20.7)	<0.001
Diastolic blood pressure, mmHg	78.92 (10.89)	78.83 (10.75)	78.46 (11.2)	79.69 (11.4)	79.30 (11.2)	<0.001
Mean arterial pressure^d^, mmHg	98.63 (12.27)	98.40 (12.03)	98.18 (12.3)	99.90 (12.9)	99.28 (12.6)	<0.001
Pulse pressure^e^, mmHg	59.13 (17.13)	58.72 (17.13)	59.16 (16.4)	60.64 (17.4)	59.93 (17.3)	<0.001

a *Hypertension was defined by (1) systolic blood pressure ≥140 mm Hg, a diastolic blood pressure ≥90 mm Hg; (2) current treatment with antihypertensive drugs in participants with a self-reported history of diagnosed hypertension*.

b *Severe hypertension was defined by systolic blood pressure ≥180 mm Hg or diastolic blood pressure ≥120 mm Hg*.

c *Awareness of hypertension was defined as the diagnosis of hypertension by a general physician or specialist*.

d *Mean arterial pressure was calculated as [(2 × diastolic blood pressure) + systolic blood pressure]/3*.

e *Pulse pressure was calculated by systolic blood pressure minus diastolic blood pressure*.

### Association Between Residential Proximity to Major Roadways and Hypertension

**Figure 2:**
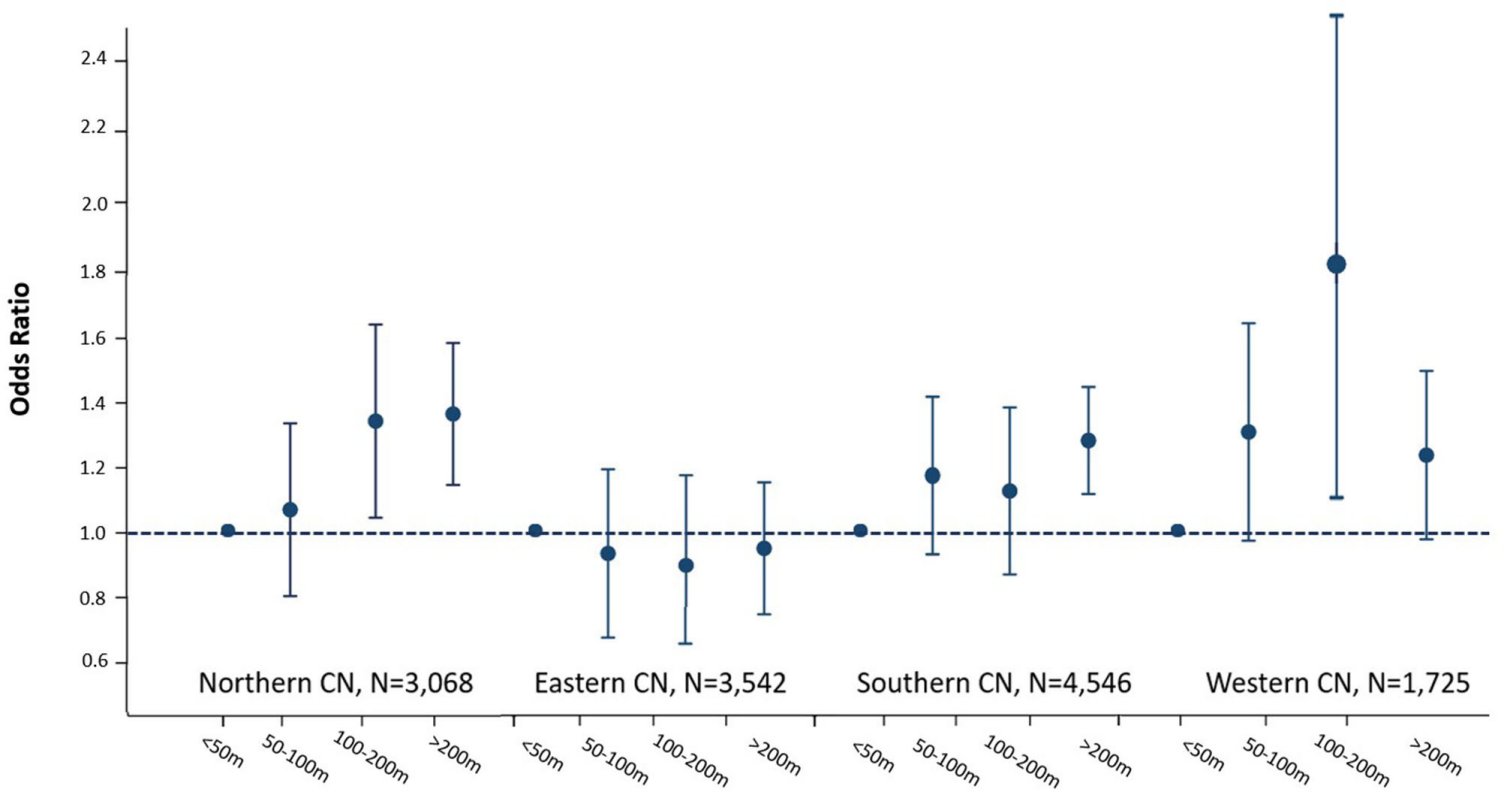
Odds ratios and 95% confidence intervals of the association between residential distance to major roadway and prevalent hypertension stratified by region. Adjustments: age at baseline, sex; adjusted for residency, education level, marital status, occupation, tobacco smoking, alcohol consumption, physical activity, body mass index, dietary diversity, appetite, intake of meat, indoor cooking ventilation, cooking fuel, family income, city population, participants' expectance of the community to provide health care education, geographical region, participation of two kinds of medical insurance (urban employee/resident medical insurance, new rural cooperative medical insurance), depressive symptom, cognitive impairment, activity of daily living, and four kinds of self-reported disease (diabetes, heart disease, stroke, and cancer). CN, China.

In Model 1 with adjustment only for sex and gender, compared with those living next to major roadways (<50 m), the odds ratio (OR) (95% CI) for those who living 50 to 100 m, 101 to 200 m, and farther than 200 m were 1.18 (1.04–1.33), 1.25 (1.09–1.44), and 1.20 (1.09–1.32), respectively. In the multivariable-adjusted logistics regression (Model 4), the associations were attenuated for those living 50 to 100 m and 101 to 200 m after additionally adjusting for health measurements and some demographic, socioeconomic, and lifestyle factors, but still significant in the groups living 100 to 200 m and living >200 m [living 50–100 m: OR = 1.17 (1.02–1.33); living 100–200 m: OR = 1.21 (1.05–1.41); living >200 m: OR = 1.22 (1.10–1.34)] (Table 3). A significant trend was observed between distance to the major roadway and hypertension (*P*s for trend <0.05 in all models). The association between residential distance to major roadway and hypertension varied significantly by study regions (Figure 2; *P* for interaction <0.001). Specifically, participants living ≥200 vs. <50 m had a 40% (95% CI = 13–74%), 25% (95% CI = 6–46%), and 29% (95% CI = 0–67%) higher odds of hypertension in the Northern, Southern, and Western China, respectively. In contrast, in Eastern China, the association was not significant (OR = 0.95, 95% CI = 0.77–1.16).

**Table 3:** Associations of residential distance to the nearest major roadway with prevalent hypertension.

**Model**	**Distance to the main traffic artery (meter), odds ratio (95% CI)**				***P* for trend**
	** <50**	**50–100**	**101–200**	**>200**	
Model 1	Ref.	1.18 (1.04–1.33)	1.25 (1.09–1.44)	1.20 (1.09–1.32)	0.030
Model 2	Ref.	1.17 (1.03–1.33)	1.22 (1.05–1.41)	1.21 (1.10–1.33)	0.009
Model 3	Ref.	1.16 (1.02–1.32)	1.21 (1.04–1.41)	1.22 (1.10–1.35)	<0.001
Model 4	Ref.	1.17 (1.02–1.33)	1.21 (1.05–1.41)	1.22 (1.10–1.34)	<0.001

*Model 1: age at baseline, sex, sampling weight; Model 2: adjusted for residency, education level, marital status, occupation, tobacco smoking, alcohol consumption, physical activity, dietary diversity, appetite, intake of meat, and body mass index. Model 3: additionally adjusted for indoor cooking ventilation, cooking fuel, family income, city population, participants' expectance of the community to provide health care education, geographical region, and participation of two kinds of medical insurance (urban employee/resident medical insurance, new rural cooperative medical insurance); Model 4: additionally adjusted for depressive symptom, cognitive impairment, activity of daily living, and four kinds of self-reported disease (diabetes, heart disease, stroke, and cancer)*.

### Association Between Residential Proximity to Major Roadways and Blood Pressure

In the age- and sex-adjusted linear regression Model 1, SBP tended to be higher among participants living more than 200 m away from the main traffic artery compared with those living within 50 m (β coefficient = 2.28, 95% CI = 1.33–3.24) (Table 4). The additional adjustment in Model 4 did not materially change the results of participants living in these four areas, but the effects were attenuated. DBP tended to be 0.79 mm Hg (95% CI = 0.25–1.32) higher among participants living more than 200 m away from the main traffic artery compared with those living within 50 m. The additional adjustment in Model 4 did not materially change the results. The linear trend ranges of the *P*-values obtained from the test of both SBP and DBP are all <0.010. In the multivariable adjusted Model 4, compared with those living 50 m from the major roadway, those living farther than 200 m had an average of 1.18 (95% CI = 0.38–1.97) mm Hg higher pulse pressure (Supplementary Table 3) and 1.00 (95% CI = 0.43–1.57) mm Hg higher mean arterial pressure (Supplementary Table 4).

**Table 4:** Association of residential distance to the major roadway with systolic and diastolic blood pressure (mm Hg).

**Model**	**Distance to the main traffic artery (meter), coefficient (95% CI)**				***P* for trend**
	** <50**	**50–100**	**101–200**	**>200**	
**Systolic blood pressure**					
Model 1	Ref.	−0.49 (−1.74 to 0.75)	−0.39 (−1.80 to 1.02)	2.28 (1.33 to 3.24)	<0.001
Model 2	Ref.	−0.19 (−1.42 to 1.04)	−0.17 (−1.57 to 1.22)	1.93 (0.98 to 2.88)	<0.001
Model 3	Ref.	0.05 (−1.17 to 1.28)	0.43 (−0.97 to 1.82)	1.85 (0.91 to 2.80)	<0.001
Model 4	Ref.	0.05 (−1.17 to 1.28)	0.33 (−1.06 to 1.72)	1.75 (0.81 to 2.70)	0.009
**Diastolic blood pressure**					
Model 1	Ref.	−0.019 (−0.69 to 0.65)	−0.34 (1.10 to 0.42)	0.87 (0.36 to 1.39)	<0.001
Model 2	Ref.	0.11 (−0.56 to 0.78)	−0.23 (−0.99 to 0.52)	0.71 (0.20 to 1.22)	<0.001
Model 3	Ref.	0.33 (−0.34 to 1.00)	0.12 (−0.64 to 0.87)	0.70 (0.18 to 1.21)	<0.001
Model 4	Ref.	0.34 (−0.33 to 1.00)	0.07 (−0.69 to 0.83)	0.70 (0.18 to 1.21)	<0.001

*Model 1: age at baseline, sex, sampling weight; Model 2: adjusted for residency, education level, marital status, occupation, tobacco smoking, alcohol consumption, physical activity, dietary diversity, appetite, intake of meat, and body mass index. Model 3: additionally adjusted for indoor cooking ventilation, cooking fuel, family income, city population, participants' expectance of the community to provide health care education, geographical region, and participation of two kinds of medical insurance (urban employee/resident medical insurance, new rural cooperative medical insurance); Model 4: additionally adjusted for depressive symptom, cognitive impairment, activity of daily living, and four kinds of self-reported disease (diabetes, heart disease, stroke, and cancer)*.

### Effect Modification

We conducted subgroup analyses by predefined factors (sex, age, residency, city population education years, physical activity, cooking fuel choice, family income, indoor cooking ventilation, ADL) (Table 5). The positive relationship between residential distance to major roadways and hypertension were generally persistent across these subgroups. Significant interactions between binary residential distance to major roadways and education level (*P* = 0.010), indoor cooking ventilation (*P* = 0.030), occupation before retirement (*P* = 0.011), and participants' expectance of the community to provide health care education (*P* = 0.028) were observed. A significantly larger effect size was observed among those participants with lower education level, manual workers, those expecting the community to provide health care education, and those who did not have any indoor ventilation compared with their counterparts.

**Table 5:** Odds ratio and 95% CI of hypertension comparing participants living in area ≥50 m vs. <50 m From the major roadway, stratified by participant characteristics.

**Characteristic**	**Odds ratio (95% CI)**,	***P* for interaction**
	**living ≥ 50 m from**	
	**the major roadway**	
Sex		0.096
Female	1.09 (0.95, 1.26)	
Male	1.27 (1.12, 1.44)	
Age, years		0.66
≥ 80	1.20 (1.067, 1.35)	
<80	1.19 (1.01, 1.39)	
Occupation before retirement		0.011
Manual and agricultural workers	1.28 (1.15, 1.43)	
White-collar workers	1.00 (0.83, 1.20)	
Residency		0.79
Urban	1.18 (1.05, 1.34)	
Rural	1.17 (1.01, 1.35)	
City population		0.69
>8 million	1.08 (0.83, 1.41)	
8 to 5 million	1.03 (0.83, 1.26)	
5 to 3 million	1.27 (1.10, 1.46)	
<3 million	1.30 (1.04, 1.64)	
Education years		0.010
<6 years	1.28 (1.15, 1.43)	
≥6 years	0.96 (0.79, 1.18)	
Physical activity		0.60
Yes	1.20 (1.07, 1.35)	
No	1.19 (1.00, 1.40)	
Cooking fuel choice		0.47
Clean	1.19 (0.99, 1.41)	
Polluted	1.27 (1.16, 1.42)	
Family income, yuan		0.80
<10,000	1.20 (1.02, 1.39)	
≥10,000	1.07 (0.95, 1.19)	
Expecting the community to		0.028
provide health care		
Yes	1.27 (1.13, 1.42)	
No	0.98 (0.81, 1.20)	
Indoor cooking ventilation		0.030
None	1.74 (1.25, 2.44)	
Mechanical or natural	1.15 (1.04, 1.26)	
Depressive symptom		0.059
With	1.05 (0.94, 1.22)	
Without	1.30 (1.12, 1.51)	
Activity of daily living		0.47
Not impaired	1.16 (1.04, 1.30)	
Impaired	1.24 (1.05, 1.48)	

*Age at baseline, sex, sampling weight, residency, education level, marital status, occupation, tobacco smoking, alcohol consumption, physical activity, body mass index, dietary diversity, appetite, intake of meat, indoor cooking ventilation, cooking fuel, family income, city population, participants' expectance of the community to provide health care education, geographical region, and participation of two kinds of medical insurance (urban employee/resident medical insurance, new rural cooperative medical insurance), depressive symptom, cognitive impairment, activity of daily living, and four kinds of self-reported disease (diabetes, heart disease, stroke, and cancer)*.

### Sensitivity Analysis

A series of sensitivity analyses were conducted to further examine the relationship between residential distance to the major roadway and hypertension by using severe hypertension as outcome (Supplementary Table 5), excluding those who took antihypertensive drug (Supplementary Table 6), those who had severe cognitive impairment (Supplementary Table 7), and those who had changed address in the last 5 year (Supplementary Table 8) and conducted the analysis by residency (Supplementary Table 9). In all sensitivity analyses, the relationship between residential proximity to major roadways and hypertension remained robust. The association between residential distance and awareness of hypertension was not significant in the multivariable adjusted Model 4 (living 50–100 m: OR = 1.01, 95% CI = 0.84–1.21; living 101–200 m: OR = 1.15, 95% CI = 0.94–1.42; living >200 m: OR = 0.96, 95% CI = 0.83–1.10) (Supplementary Table 10).

## Discussion

In this nationally representative population-based cohort including 12,881 Chinese adults 65 years or older, we evaluated the association between residential proximity to major roadways and prevalence of hypertension. Our principal finding was that living closer to major roadways was associated with lower odds of hypertension. Compared to those living beyond 200 m from major roadways, the odds of cognitive impairments were 20% lower for those living within 50 m from major roadways, independent of other risk factors. We observed significant effect modifications from SES and accessibility to health care resources, indicating that the inequity of accessibility to health services may partly modify the associations. The significance of the observed associations varied considerably across study regions, with positive associations observed in the Northern, Southern, and Western China, whereas there was no significant association observed in the Eastern China.

In previous studies, the association of residential distance to major roadways with hypertension was reported but with inconsistent results in populations living in developed countries (10, 23). Among 4,291 participants of the Heinz Nixdorf Recall Study in Germany, the association of volumes of heavy-duty traffic and prevention of hypertension was not statistically significant (5, 24). The study also reported that cumulative traffic density within 100 m from home was not associated with prevalent hypertension in a pooled analysis of 13 European cohorts with a combined sample size of more than 100,000 participants (10). In a study of 5,401 Women's Health Initiative (WHI) participants in the San Diego area, those living within 100 m from a major roadway had a 22% higher risk of hypertension (23). Another similar longitudinal study including 38,360 WHI participants with a median follow-up of 7.9 years found that living close to major roadways was associated with higher risk of hypertension, and this association varied by different study sites in the United States, with pronounced associations in the West and Northeast, a reverse association in the South, and a non-significant association in the Midwest (6). This discrepancy between our results and those obtained from studies conducted in developed countries may stem from the different demographic characteristics along the different distances to major roadways. To be more specific, in those previous studies conducted in populations in developed countries, individuals living near major roadways have lower SES and lower education levels, and it is possible that the observed beneficial effects of living “farther from major roadways may be confounded by potential covariates (23, 25). In our study, we did not observe substantial differences in levels of income or education along the different distances to major roadways, but we found a significantly higher level of physical activity among those living close to major roadways. A higher level of physical activity was proven to be associated with a lower incidence rate of hypertension (26), which may be one potential explanation for the discrepancy between our results and those from the studies conducted in developed countries.

We evaluated whether the association between residential distance to a major roadway and hypertension varied across strata defined by individual-level characteristics, and we found statistically significant heterogeneity by education, occupation history, expectance of the community to provide health care, health education, and indoor cooking ventilation. Generally, the associations were more pronounced in those who had lower SES and poor availability of health care services, suggesting that the inequity in accessibility to health care resources may mediate the associations. On the other hand, it may also lead to the inconsistency between our results and those from the studies conducted in developed countries. Our sample had a lower education level (>6 years of schooling: 23.1%) and limited access to community-wide medical resources, compared with populations in developed countries (27).

In the present study, a considerable level of heterogeneity by different geographical regions was observed, with negative associations demonstrated in the Northern, Southern, and Western China, whereas there was no association shown in the Eastern China. The difference in levels of traffic-related exposure may be one potential explanation. In Kingsley et al. study, they hypothesized that the difference in traffic-related air pollution across different regions of the United States may lead to the variation of this association (6). In our study, we also found that the association of residential distance to a major roadway with hypertension was not significant in Eastern China, the most affluent region in China, and this level of affluence may indicate a higher level of vehicle ownerships, as well as more developed and advanced infrastructure of public transportation in this region. Normally, distance to a major roadway is regarded as a proxy for the residential exposure to traffic-related air and noise pollution in previous cardiovascular studies. However, living near major roadways is also a multifactor exposure that includes, apart from high levels of exposure to air and noise pollution, other factors such as different SES or living arrangements. It is plausible that the risk of high levels of traffic-related exposure including air and noise pollution near major roadways may be dominated by the beneficial effects from other socioeconomic factors. In addition to the traffic-related exposure, a number of other location-specific characteristics, such as the distribution of medical resources and living arrangements, may increase the variability across regions.

The use of residential proximity to major roadways as a potential risk factor for hypertension has several significant advantages. First, it allows for the consideration of the joint effects of air and noise pollution, as well as other socioeconomic factors originating from this ubiquitous source. Second, it is easy to measure and contemplate relevant further policy implications. For instance, for those policymakers, it might be beneficial to allocate more public medical resources such as community hospital or the clinic in the center of a community. Our study is the first to demonstrate its association with hypertension in LMICs, but we revealed a different association, which indicates that this association with hypertension might vary by different countries or different regions within the same country. Such level of variability points to and validates the necessity of establishing said association on a country by country/region-by-region basis. The utility of residential proximity to major roadways as a novel marker for higher risks of hypertension may need to be reconsidered and need to be revisited in other LMICs. Additionally, it is possible that the association between residential distances to major roadways and cardiovascular diseases in LMICs might also be different from that in developed countries.

Our study has some limitations. First, we cannot rule out the possibility that participants with hypertension are more likely to choose to live closer to major roadways because of its cross-sectional design. However, our results remained robust when adjusted for a number of individual-level indicators of SES to account for the potential confounding effects. However, prospective studies of incidences of hypertension are needed to completely rule out such possibility. Second, we use self-reported distances to major roadways as an approximation for the exactly measured distances. However, in order to reduce the recall bias, we have conducted sensitivity analyses by excluding those with severe cognitive impairments, and the results remained robust. Although such approximation can be inaccurate to some degree, it can help classify the living distances to major roadways into various categories and separate those who live near major roadways from those who do not. Third, because of the cross-sectional design of most major roadways, we could not evaluate the long-term impact of living near them on hypertension, but we conducted a sensitivity analysis by excluding those participants who have changed their addresses in the last 5 years. Lastly, we did not have better ways to measure the accessibility to medical resources; however, we used the participants' expectation to health care and health education to roughly gauge the medical resource accessibility. Our study has vital strengths, including large sample size, diversity in geographical regions and SES, and the robustness to adjust for many potential confounders.

## Conclusion

Our results suggest that among the population older than 65 years in China, living close to major roadways is associated with lower odds of hypertension, with varied significance of association across different regions. These results indicate that the association of residential distances to major roadways, a multilevel exposure, with hypertension needs to be reexamined in LMICs. Moreover, the importance of traffic-related exposure such as noise or air pollution compared with socioeconomic factors also needs to be revisited in those countries. Our results, if confirmed as causal in further longitudinal studies, pave way for an evidence-based policy-level intervention to reduce the burden of hypertension more effectively and efficiently as the cardiovascular diseases and aging processing have begun for LMICs.

## Data Availability Statement

The data, analytical methods, and study materials will be made available to other researchers for purposes of reproducing the results or replicating the procedure from the corresponding author on reasonable request or the Peking University Open Research Data plantform.

## Ethics Statement

The studies involving human participants were reviewed and approved by Peking University and Duke University. The patients/participants provided their written informed consent to participate in this study.

## Author Contributions

YY and XJ: conceptualization, methodology, data analysis, and writing. KC, KZ, and TZ: methodology, validation, and writing. DY and HZ: validation, writing with review, and editing. JZ: conceptualization, methodology, data analysis, writing with review, and editing. YY and YZ: conceptualization, funding acquisition, project administration, writing with review, and editing. All authors contributed to the article and approved the submitted version.

## Conflict of Interest

The authors declare that the research was conducted in the absence of any commercial or financial relationships that could be construed as a potential conflict of interest.
