# Supplementary material for: Residential Proximity to Major Roadways and Prevalent Hypertension Among Older Women and Men: Results From the Chinese Longitudinal Healthy Longevity Survey
Source: Front Cardiovasc Med. 2020 Nov 17;7:587222. doi: 10.3389/fcvm.2020.587222 (PMC7705226; doi:10.3389/fcvm.2020.587222)
Supplement: Supplementary file 1 [file Data_Sheet_1.PDF]

Appendix Supplementary Files

Residential Proximity to Major Roadways and Prevalent Hypertension Among Older women and men: Results from the Chinese Longitudinal Healthy Longevity Survey

Yao Yao, Kaixi Cao, Kehan Zhang, Tinglong Zhu, Dahai Yue, Hao Zhang, Jim Zhang*, Xurui Jin*, Yi Zeng*

Table S1.. **Page 2**
Characteristics of 12,881 participants aged 65 years old and older in Chinese Longitudinal Healthy Longevity Survey.

Table S2.. **Page 4**
Characteristics of the Analyzed Sample and Total Sample

Table S3.. **Page 6**
Association of Residential Distance to the Major Roadway with Pulse Pressure (mm Hg).

Table S4.. **Page 7**
Association of Residential Distance to the Major Roadway with Mean Arterial Pressure (mm Hg).

Table S5.. **Page 8**
Sensitivity Analysis of Association of Residential Distance to Major Roadway with Prevalent Hypertension by using Severe Hypertension as outcome.

Table S6.. **Page 9**
Sensitivity Analysis of Association of Residential Distance to Major Roadway with Prevalent Hypertension by excluding those who Took Anti-hypertensive Drug.

Table S7.. **Page 10**
Sensitivity Analysis of Association of Residential Distance to Major Roadway with Prevalent Hypertension by excluding the Participants with Severe Cognitive Impairment.

Table S8.. **Page 11**
Sensitivity Analysis of Association of Residential Distance to Major Roadway with Prevalent Hypertension by excluding the Participants who have Changed their Residential Address in Five Years.

Table S9.. **Page 12**
Sensitivity Analysis of Association of Residential Distance to Major Roadway with Prevalent Hypertension by Replicating the Analysis by Residency.

Table S10.. **Page 13**
Association of Residential Distance to Major Roadway with Awareness of Hypertension.

Table S1. **Characteristics of 12,881 participants aged 65 years old and older in Chinese Longitudinal Healthy Longevity Survey.**

Characters	Distance to the major roadway			*P*-value
	<50	≥50	Total	
**N**	2352	10529	12,881	
**Age**, years, Mean (SD)	84.6 (11.6)	85.4 (11.8)	85.2 (11.7)	0.041
**Sex**, Count (%)				0.65
Male	1050 (44.6)	4647 (44.1)	5,697 (44.2)	
Female	1302 (55.4)	5882 (55.9)	7,184 (55.8)	
**Education**, Count (%)				0.42
None (0 year)	1342 (57.1)	6060 (57.6)	7,402 (57.5)	
Primary school (1-6 years)	443 (18.8)	2056 (19.5)	2,499 (19.4)	
Middle school or higher (>6 years)	567 (24.1)	2413 (22.9)	2,980 (23.1)	
**Occupation before retirement**, Count (%)				0.50
Manual and agricultural workers	1696 (72.1)	7520 (71.4)	9,250 (71.8)	
White-collar workers	656 (27.9)	3009 (28.6)	3,631 (28.2)	
**Hypertension** [a], Count (%)				<0.001
Without	935 (39.8)	3814 (36.2)	4,749 (36.9)	
With	1417 (60.2)	6715 (63.8)	8,132 (63.1)	
**Residence**, Count (%)				<0.001
Urban	977 (41.5)	4886 (46.4)	7,021 (54.5)	
Rural	1375 (58.5)	5643 (53.6)	5,860 (45.5)	
**Marital status**, Count (%)				0.08
Currently married and living with spouse	1381 (58.7)	6388 (60.7)	7,769 (60.3)	
Others [b]	971 (41.3)	4141 (39.3)	5,112 (39.7)	
**Dietary diversity** [c], Count (%)				0.70
High	1808 (76.9)	8054 (76.5)	9,854 (76.5)	
Low	544 (23.1)	2475 (23.5)	3,027 (23.5)	
**Tobacco Smoking**, Count (%)				0.14
Current	1974 (83.9)	8964 (85.1)	1,943 (15.1)	
Not current	378 (16.1)	1565 (14.9)	10,938 (84.9)	
**Alcohol consumption**, Count (%)				0.40
Current	2029 (86.3)	9012 (85.6)	1,840 (14.3)	
Not current	323 (13.7)	1517 (14.4)	11,041 (85.7)	
**Physical activity**, Count (%)				<0.001
Yes	1545 (65.7)	7349 (69.8)	3,987 (31.0)	
No	807 (34.3)	3180 (30.2)	8,894 (69.0)	
**Appetite,** Count (%)				0.11
Salty	1865 (79.3)	8500 (80.7)	10,365 (80.3)	
Others [d]	487 (20.7)	2029 (19.3)	2,516 (19.7)	
**Meat consumption,** Count (%)				0.82
Almost every day	1793 (77.3)	8070 (77.5)	9,863 (77.1)	
Rarely or never	527 (22.7)	2343 (22.5)	2,870 (22.9)	
**Fuel choice**, Count (%)				<0.001
Clean	1728 (73.5)	7287 (69.2)	9,008 (69.9)	
Polluted	624 (26.5)	3242 (30.8)	3,873 (30.1)	
**Family annual income,** Count (%)				0.31
≥30,000	1369 (58.2)	6008 (57.1)	7,377 (57.3)	
<30,000	983 (41.8)	4521 (42.9)	5,504 (42.7)	
**Indoor cooking ventilation**, Count (%)				0.57
None	199 (8.5)	929 (8.8)	1,128(8.7)	

Mechanical or window	2153 (91.5)	9600 (91.2)	11,753 (91.3)	
**BMI (kg/m²),** Mean (SD)	22.4 (3.9)	22.2 (3.8)	22.3 (3.9)	<0.001
**City Population**, Count (%)				<0.001
>8 million	419 (17.9)	2224 (21.2)	2,643 (20.5)	
8 to 5 million	1107 (47.2)	4984 (47.5)	6,091 (47.3)	
5 to 3 million	503 (21.4)	1897 (18.1)	2,400 (18.6)	
<3 million	317 (13.5)	1398 (13.3)	1,715 (13.3)	
**Geographical Region** [e]				0.094
Northern China	493 (21.0)	2575 (24.5)	3,068 (23.8)	
Eastern China	581 (24.8)	2929 (27.9)	3,510 (27.2)	
Southern China	919 (39.2)	3627 (34.5)	4,546 (35.3)	
Western China	353 (15.0)	1372 (13.1)	1,725 (13.4)	
**Urban employee/resident medical insurance**, Count (%)				0.25
Have	559 (23.8)	2343 (22.3)	2,892 (22.5)	
Do not have	1793 (76.2)	8186 (77.7)	9,979 (77.5)	
**New rural cooperative medical insurance**, Count (%)				0.25
Have	1464 (62.2)	6686 (63.5)	8,150 (63.5)	
Do not have	888 (37.8)	3843 (36.5)	4,731 (36.5)	
**Expectance of the community to provide health care education**, Count (%)				0.67
Yes	1764 (75.0)	7817 (74.2%)	9,581 (74.3)	
No	588 (25.0)	2712 (25.8%)	3,300 (25.6)	
**Cognitive impairment** [f], Count (%)				0.85
No cognitive impairment	1422 (60.5)	6343 (60.2)	7,776 (60.4)	
With cognitive impairment	930 (39.5)	4186 (39.8)	5,105 (39.6)	
**Activity of daily living**, Count (%)				0.18
No activity of daily living	1647 (70.0)	7520 (71.4)	9,167 (71.2)	
With activity of daily living	705 (30.0)	3009 (28.6)	3,714 (28.8)	
**Depressive symptom** [g], Count (%)				0.077
No depressive symptom	1072 (45.6)	4588 (43.6)	5,672 (44.0)	
With depressive symptom	1280 (54.4)	5941 (56.4)	7,209 (56.0)	

a: hypertension was defined by systolic blood pressure≥140 mm Hg or a diastolic blood pressure≥90 mm Hg.

b: Others' include widowed, separated, divorced, and never married.

c: Dietary diversity was calculated by intake frequency of eight food (vegetables, fruits, legumes and their products, nuts, meat, eggs, fish, dairy and its products) and dichotomized as high vs. low with the cutoff of 6

d: Others include bland, sweet, spicy and prefer raw food.

e: Northern China includes Jilin, Liaoning, Heilongjiang, Beijing, Tianjin, Shanxi, Hebei, and Shandong province. Eastern China includes Shanghai, Anhui, Shanxi, Zhejiang, Fujian, and Jiangsu province. Southern China includes Guangdong, Guangxi, Henan, Hainan, Hubei, and Hunan province. Western China includes Sichuan, Chongqing, and Shaanxi province.

f: Cognitive impairment was defined as MMSE score lower to 24.

g: Depressive symptom was defined by Center for Epidemiologic Studies Depression Scale score equal to or higher than 10.

Table S2. **Characteristics of the Analyzed Sample and Total Sample.**

Characters	Analyzed sample (N = 12,881)	Total sample (N = 15,738)	*P*-value
**Age**, years, Mean (SD)	85.2 (11.7)	85.4 (11.7)	0.131
**Sex**, Count (%)			0.274
Male	5,697 (44.2)	6859 (43.6)	
Female	7,184 (55.8)	8879 (56.4)	
**Education**, Count (%)			0.352
None (0 year)	7,402 (57.5)	9105 (57.9)	
Primary school (1-6 years)	2,499 (19.4)	2948 (18.7)	
Middle school or higher (>6 years)	2,980 (23.1)	3685 (23.4)	
**Occupation before retirement**, Count (%)			0.182
Manual and agricultural workers	3,631 (28.2)	11147 (70.8)	
White-collar workers	9,250 (71.8)	4591 (29.2)	
**Residence**, Count (%)			0.095
Urban	5,860 (45.5)	7008 (44.5)	
Rural	7,021 (54.5)	8730 (55.5)	
**Marital status**, Count (%)			0.067
Currently married and living with spouse	7769 (60.3)	9659 (61.4)	
Others [b]	5112 (39.7)	6079 (38.6)	
**Dietary diversity** [c], Count (%)			0.974
High	9,854 (76.5)	12052 (76.6)	
Low	3,027 (23.5)	3686 (23.4)	
**Tobacco Smoking**, Count (%)			0.195
Current	10938 (84.9)	13450 (85.5)	
Not current	1943 (15.1)	2288 (14.5)	
**Alcohol consumption**, Count (%)			0.302
Current	11041 (85.7)	13557 (86.1)	
Not current	1840 (14.3)	2181 (13.9)	
**Physical activity**, Count (%)			0.003
Yes	8894 (69.0)	11122 (70.7)	
No	3987 (31.0)	4616 (29.3)	
**Appetite,** Count (%)			0.048
Salty	10365 (80.5)	12809 (81.4)	
Others [d]	2516 (19.5)	2929 (18.6)	
**Meat consumption,** Count (%)			0.459
Almost every day	9863 (77.5)	11904 (77.1)	
Rarely or never	2870 (22.5)	3538 (22.9)	
**Fuel choice**, Count (%)			0.662
Clean	9,008 (69.9)	11052 (70.2)	
Polluted	3,873 (30.1)	4686 (29.8)	
**Family annual income,** Count (%)			0.348
≥30,000	7,377 (57.3)	9100 (57.8)	
<30,000	5,504 (42.7)	6638 (42.2)	

**BMI (kg/m²),** Mean (SD)	22.3 (3.9)	22.2 (3.9)	0.72
**City Population**, Count (%)			0.016
>8 million	2,643 (20.5)	3411 (21.7)	
8 to 5 million	6,091 (47.3)	7179 (45.7)	
5 to 3 million	2,400 (18.6)	3030 (19.3)	
<3 million	1,715 (13.3)	2075 (13.2)	
**Geographical Region** [e]			0.147
Northern China	3,068 (23.8)	3604 (23.0)	
Eastern China	3,510 (27.2)	4426 (28.2)	
Southern China	4,546 (35.3)	5610 (35.7)	
Western China	1,725 (13.4)	2055 (13.1)	
**Urban employee/resident medical insurance**, Count (%)			0.031
Have	2902 (22.5)	3716 (23.6)	
Do not have	9979 (77.5)	12022 (76.4)	
**New rural cooperative medical insurance**, Count (%)			0.212
Have	8150 (63.3)	9845 (62.6)	
Do not have	4731 (36.7)	5893 (37.4)	
**Expectance of the community to provide health care education**, Count (%)			0.004
Yes	7,776 (60.4)	9771 (64.4)	
No	5,105 (39.6)	5396 (35.6)	
**Activity of daily living**, Count (%)			0.040
No activity of daily living	9,167 (71.2)	11025 (70.1)	
With activity of daily living	3,714 (28.8)	4713 (29.9)	
**Depressive symptom** [g], Count (%)			<0.001
No depressive symptom	5,672 (44.0)	6584 (41.8)	
With depressive symptom	7,209 (56.0)	9154 (58.2)	

a: hypertension was defined by systolic blood pressure≥140 mm Hg or a diastolic blood pressure≥90 mm Hg.
b: Others' include widowed, separated, divorced, and never married.
c: Dietary diversity was calculated by intake frequency of eight food (vegetables, fruits, legumes and their products, nuts, meat, eggs, fish, dairy and its products) and dichotomized as high vs. low with the cutoff of 6.
d: Others include bland, sweet, spicy and prefer raw food.
e: Northern China includes Jilin, Liaoning, Heilongjiang, Beijing, Tianjin, Shanxi, Hebei, and Shandong province. Eastern China includes Shanghai, Anhui, Shanxi, Zhejiang, Fujian, and Jiangsu province. Southern China includes Guangdong, Guangxi, Henan, Hainan, Hubei, and Hunan province. Western China includes Sichuan, Chongqing, and Shaanxi province.
f: Cognitive impairment was defined as MMSE score lower to 24.
g: Depressive symptom was defined by Center for Epidemiologic Studies Depression Scale score equal to or higher than 10.

Table S3. **Association of Residential Distance to the Major Roadway with Pulse Pressure (mm Hg).**

Model	Distance to the main traffic artery (meter), coefficient (95% CI)				*P* for trend
	**<50**	**50-100**	**101-200**	**>200**	
**Pulse Pressure**					
Model 1	Ref.	-0.47 (-1.51, 0.56)	-0.05 (-1.23, 1.12)	1.41 (0.61, 2.21)	<0.001
Model 2	Ref.	-0.28 (-1.32, 0.76)	0.07 (-1.11, 1.24)	1.22 (0.43, 2.02)	<0.001
Model 3	Ref.	-0.24 (-1.27, 0.80)	0.26 (-0.92, 1.43)	1.29 (0.49, 2.08)	<0.001
Model 4	Ref.	-0.25 (-1.29, 0.78)	0.22 (-0.95, 1.39)	1.18 (0.38, 1.97)	0.009

Adjustments: Model 1: age at baseline, sex, sampling weight; Model 2: adjust for residency, education level, marital status, occupation, tobacco smoking, alcohol consumption, physical activity, dietary diversity, appetite, intake of meat and body mass index. Model 3: additionally adjust for indoor cooking ventilation, cooking fuel, family income, city population, participants' expectance of the community to provide health care education, geographical region, participation of two kinds of medical insurance (Urban employee/resident medical insurance, New rural cooperative medical insurance); Model 4: additionally adjust for depressive symptom, cognitive impairment, activity of daily living and four kinds of self-reported disease (diabetes, heart disease, stroke, and cancer).

Table S4. **Association of Residential Distance to the Major Roadway with Mean Arterial Pressure (mm Hg).**

Model	Distance to the main traffic artery (meter), coefficient (95% CI)				*P* for trend
	**<50**	**50-100**	**101-200**	**>200**	
**Mean Arterial Pressure**					
Model 1	Ref.	-0.17 (-0.94, 0.58)	-0.37 (-1.22, 0 .50)	1.34 (0.76, 1.93)	<0.001
Model 2	Ref.	-0.20 (-0.55, 0.95)	-0.03 (-0.88, 0.82)	1.11 (0.54,1.69)	<0.001
Model 3	Ref.	0.30 (-0.44, 1.05)	0.12 (-0.73, 0.96)	1.05 (0.48, 1.63)	<0.001
Model 4	Ref.	0.29 (-0.45,1.04)	0.04 (-0.81, 0.89)	1.00 (0.43, 1.57)	0.009

Adjustments: Model 1: age at baseline, sex, sampling weight; Model 2: adjust for residency, education level, marital status, occupation, tobacco smoking, alcohol consumption, physical activity, dietary diversity, appetite, intake of meat and body mass index. Model 3: additionally adjust for indoor cooking ventilation, cooking fuel, family income, city population, participants' expectance of the community to provide health care education, geographical region, participation of two kinds of medical insurance (Urban employee/resident medical insurance, New rural cooperative medical insurance); Model 4: additionally adjust for depressive symptom, cognitive impairment, activity of daily living and four kinds of self-reported disease (diabetes, heart disease, stroke, and cancer).

Table S5. **Sensitivity Analysis of Association of Residential Distance to Major Roadway with Prevalent Hypertension by using Severe Hypertension as outcome.**

Model	Distance to the main traffic artery (meter), odds ratio (95% CI)				*P* for trend
	**<50**	**50-100**	**101-200**	**>200**	
Model 1	Ref.	0.93 (0.79, 1.11)	0.91 (-0.75, 1.11)	1.22 (1.08, 1.39)	<0.001
Model 2	Ref.	0.94 (0.79, 1.1)	0.92 (0.75, 1.11)	1.22 (1.07, 1.38)	<0.001
Model 3	Ref.	0.98 (0.82, 1.16)	0.99 (0.81, 1.21)	1.21 (1.07, 1.38)	<0.001
Model 4	Ref.	0.98 (0.82, 1.17)	0.99 (0.81, 1.20)	1.21 (1.06, 1.38)	<0.001

Adjustments: Model 1: age at baseline, sex, sampling weight; Model 2: adjust for residency, education level, marital status, occupation, tobacco smoking, alcohol consumption, physical activity, dietary diversity, appetite, intake of meat and body mass index. Model 3: additionally adjust for indoor cooking ventilation, cooking fuel, family income, city population, participants' expectance of the community to provide health care education, geographical region, participation of two kinds of medical insurance (Urban employee/resident medical insurance, New rural cooperative medical insurance); Model 4: additionally adjust for depressive symptom, cognitive impairment, activity of daily living and four kinds of self-reported disease (diabetes, heart disease, stroke, and cancer).

Table S6. **Sensitivity Analysis of Association of Residential Distance to Major Roadway with Prevalent Hypertension by excluding those who took Anti-hypertensive Drug.**

Model	**Distance to the main traffic artery (meter),** odds ratio (95% CI)				*P* for trend
	**≤50**	**50-100**	**101-200**	**>200**	
Model 1	Ref.	1.13 (1.00, 1.28)	1.24 (1.07, 1.41)	1.17 (1.07, 1.29)	0.003
Model 2	Ref.	1.10 (0.97, 1.26)	1.21 (1.04, 1.43)	1.16 (1.05, 1.28)	0.011
Model 3	Ref.	1.09 (0.95, 1.24)	1.22 (1.05, 1.42)	1.17 (1.05, 1.30)	0.016
Model 4	Ref.	1.09 (0.96, 1.25)	1.22 (1.05, 1.43)	1.16 (1.05, 1.29)	0.314

Adjustments: Model 1: age at baseline, sex, sampling weight; Model 2: adjust for residency, education level, marital status, occupation, tobacco smoking, alcohol consumption, physical activity, dietary diversity, appetite, intake of meat and body mass index. Model 3: additionally adjust for indoor cooking ventilation, cooking fuel, family income, city population, participants' expectance of the community to provide health care education, geographical region, participation of two kinds of medical insurance (Urban employee/resident medical insurance, New rural cooperative medical insurance); Model 4: additionally adjust for depressive symptom, cognitive impairment, activity of daily living and four kinds of self-reported disease (diabetes, heart disease, stroke, and cancer).

Table S7. **Sensitivity Analysis of Association of Residential Distance to Major Roadway with Prevalent Hypertension by excluding the participants with Severe Cognitive Impairment*.**

Model	**Distance to the Major Roadway (meter), odds ratio (95% CI)**				*P* for trend
	**≤50**	**50-100**	**101-200**	**>200**	
Model 1	Ref.	1.17 (1.03, 1.34)	1.26 (1.09, 1.46)	1.18 (1.08, 1.31)	0.003
Model 2	Ref.	1.17 (1.03, 1.33)	1.24 (1.07, 1.45)	1.20 (1.09, 1.33)	0.011
Model 3	Ref.	1.14 (1.01, 1.31)	1.23 (1.07, 1.44)	1.20 (1.09, 1.33)	0.016
Model 4	Ref.	1.15 (1.01, 1.31)	1.24 (1.07, 1.44)	1.19 (1.08, 1.32)	0.314

Adjustments: Model 1: age at baseline, sex, sampling weight; Model 2: adjust for residency, education level, marital status, occupation, tobacco smoking, alcohol consumption, physical activity, dietary diversity, appetite, intake of meat and body mass index. Model 3: additionally adjust for indoor cooking ventilation, cooking fuel, family income, city population, participants' expectance of the community to provide health care education, geographical region, participation of two kinds of medical insurance (Urban employee/resident medical insurance, New rural cooperative medical insurance); Model 4: additionally adjust for depressive symptom, cognitive impairment, activity of daily living and four kinds of self-reported disease (diabetes, heart disease, stroke, and cancer).

*Severe cognitive impairment was defined by MMSE score lower than 19.

Table S8. **Sensitivity Analysis of Association of Residential Distance to Major Roadway with Prevalent Hypertension by excluding the Participants who have Changed their Residential Address in Five Years.**

Model	Distance to the Major Roadway (meter), odds ratio (95% CI)				*P* for trend
	**≤50**	**50-100**	**101-200**	**>200**	
Model 1	Ref.	1.18 (1.04, 1.34)	1.28 (1.11, 1.48)	1.20 (1.09, 1.32)	0.003
Model 2	Ref.	1.18 (1.04, 1.34)	1.26 (1.09, 1.46)	1.21 (1.10, 1.34)	0.011
Model 3	Ref.	1.16 (1.02, 1.32)	1.25 (1.08, 1.46)	1.21 (1.10, 1.34)	0.016
Model 4	Ref.	1.15 (1.01, 1.31)	1.25 (1.08, 1.45)	1.20 (1.09, 1.33)	0.314

Adjustments: Model 1: age at baseline, sex, sampling weight; Model 2: adjust for residency, education level, marital status, occupation, tobacco smoking, alcohol consumption, physical activity, dietary diversity, appetite, intake of meat and body mass index. Model 3: additionally adjust for indoor cooking ventilation, cooking fuel, family income, city population, participants' expectance of the community to provide health care education, geographical region, participation of two kinds of medical insurance (Urban employee/resident medical insurance, New rural cooperative medical insurance); Model 4: additionally adjust for depressive symptom, cognitive impairment, activity of daily living and four kinds of self-reported disease (diabetes, heart disease, stroke, and cancer).

Table S9. **Sensitivity Analysis of Association of Residential Distance to Major Roadway with Prevalent Hypertension by Replicating the Analysis by Residency.**

	**Distance to the Major Roadway (meter), odds ratio (95% CI)**			
	**≤50**	**50-100**	**101-200**	**>200**
Rural	Ref.	1.16 (0.94, 1.44)	124 (0.97, 1.58)	1.19 (1.02, 1.39)
Urban	Ref.	1.15 (0.97, 1.36)	1.20 (1.00, 1.45)	1.21 (1.06, 1.38)

Adjustments: Model 1: age at baseline, sex, sampling weight; Model 2: adjust for residency, education level, marital status, occupation, tobacco smoking, alcohol consumption, physical activity, dietary diversity, appetite, intake of meat and body mass index. Model 3: additionally adjust for indoor cooking ventilation, cooking fuel, family income, city population, participants' expectance of the community to provide health care education, geographical region, participation of two kinds of medical insurance (Urban employee/resident medical insurance, New rural cooperative medical insurance); Model 4: additionally adjust for depressive symptom, cognitive impairment, activity of daily living and four kinds of self-reported disease (diabetes, heart disease, stroke, and cancer).

Table S10. **Association of Residential Distance to Major Roadway with Awareness of Hypertension.**

	**Distance to the Major Roadway (meter),** odds ratio (95% CI)			
	**≤50**	**50-100**	**101-200**	**>200**
Model 1	Ref.	1.13 (0.96, 1.34)	1.36 (1.13, 1.65)	0.89 (0.78, 1.02)
Model 2	Ref.	1.03 (0.86, 1.22)	1.24 (1.02, 1.52)	0.98 (0.86, 1.12)
Model 3	Ref.	1.01 (0.84, 1.20)	1.14 (0.93, 1.40)	0.97 (0.84, 1.11)
Model 4	Ref.	1.01 (0.84, 1.21)	1.15 (0.94, 1.42)	0.96 (0.83, 1.10)

Adjustments: Model 1: age at baseline, sex, sampling weight; Model 2: adjust for residency, education level, marital status, occupation, tobacco smoking, alcohol consumption, physical activity, dietary diversity, appetite, intake of meat and body mass index. Model 3: additionally adjust for indoor cooking ventilation, cooking fuel, family income, city population, participants' expectance of the community to provide health care education, geographical region, participation of two kinds of medical insurance (Urban employee/resident medical insurance, New rural cooperative medical insurance); Model 4: additionally adjust for depressive symptom, cognitive impairment, activity of daily living and four kinds of self-reported disease (diabetes, heart disease, stroke, and cancer).
